# Hypotheses on Atherogenesis Triggering: Does the Infectious Nature of Atherosclerosis Development Have a Substruction?

**DOI:** 10.3390/cells12050707

**Published:** 2023-02-23

**Authors:** Konstantin A. Lusta, Anastasia V. Poznyak, Vasily N. Sukhorukov, Ilya I. Eremin, Irina I. Nadelyaeva, Alexander N. Orekhov

**Affiliations:** 1 Institute for Atherosclerosis Research, Osennyaya 4-1-207, 121609 Moscow, Russia; 2 Laboratory of Angiopathology, Institute of General Pathology and Pathophysiology, 8 Baltiiskaya Street, 125315 Moscow, Russia; 3 Petrovsky National Research Centre of Surgery, 2, Abrikosovsky Lane, 119991 Moscow, Russia

**Keywords:** atherosclerosis, cardiovascular disease, inflammation, infections, lipoprotein modification, endothelial dysfunction, pathogen-associated molecular patterns, cytokines

## Abstract

Since the end of the 20th century, it has been clear that atherosclerosis is an inflammatory disease. However, the main triggering mechanism of the inflammatory process in the vascular walls is still unclear. To date, many different hypotheses have been put forward to explain the causes of atherogenesis, and all of them are supported by strong evidence. Among the main causes of atherosclerosis, which underlies these hypotheses, the following can be mentioned: lipoprotein modification, oxidative transformation, shear stress, endothelial dysfunction, free radicals’ action, homocysteinemia, diabetes mellitus, and decreased nitric oxide level. One of the latest hypotheses concerns the infectious nature of atherogenesis. The currently available data indicate that pathogen-associated molecular patterns from bacteria or viruses may be an etiological factor in atherosclerosis. This paper is devoted to the analysis of existing hypotheses for atherogenesis triggering, and special attention is paid to the contribution of bacterial and viral infections to the pathogenesis of atherosclerosis and cardiovascular disease.

## 1. Introduction

The most common heart diseases are prompted by atherosclerosis (AS). Thus, ischemia is caused by insufficient blood supply to the heart muscle. Oxygen starvation occurs when the lumen narrows as a result of plaque formation on the arteries’ inner walls. Lipids, cholesterol, calcium, and fibrin are deposited in atherosclerotic plaques [1]. These deposits block the blood flow, leading to thrombosis, heart attack, or stroke. Cardiovascular diseases (CVD) are the leading cause of death worldwide [2], and the main CVD contributor is AS of the coronary and other large arteries [3]. The main AS manifestations are damage to the vascular endothelial cells (VECs) of the vessel walls; recruitment of leukocytes and monocytes, with their subsequent transformation into macrophages in the vascular subendothelium; absorption of lipids by macrophages; the formation of fatty streaks; and calcification and fibrinization of the intimal layer. All these pathological changes in blood vessels lead to myocardial infarction and stroke [4].

AS is a chronic inflammatory disease, suggesting that various infections play an important role in its development. Many studies provide pieces of evidence that many different pathogens detected in atherosclerotic lesions initiate a cascade of inflammatory processes and accelerate plaque growth in the blood vessel walls [5]. The stressor effect of bacterial and viral infection on the vascular wall is the most substantial known risk factor [6]. The suggestions of the possibility of pathogenic bacteria and/or viruses’ involvement in AS pathogenesis are encouraged by coincidences between the CVD morbidity and infection markers; moreover, another piece of evidence is an enhanced rate of atherogenesis at pathogen infection [7].

## 2. Hypotheses on the Principles for Triggering Atherogenesis

### 2.1. Inflammation

The leading cause behind AS triggering has not yet been clearly established. There are many hypotheses on the possible causes of pathological process development. First of all, inflammation plays a key role in atherogenesis and is involved in every stage of this pathogenesis. The inflammatory nature of AS is now firmly established and is quite well studied. The driving forces behind inflammation are endothelial dysfunction, altered lipoprotein metabolism, hemodynamic shear stress, free radicals, hypertension, diabetes mellitus, genetic alterations, elevated level of homocysteine, infectious microorganisms, and viruses. Notably, the most probable scenario could be the combination of all these or other factors [4,8]. Inflammation operates as a common basis for atherogenesis and progression. In the blood vessel walls, inflammation is accompanied by the release of pro-inflammatory cytokines and chemokines, bioactive lipoproteins, adhesion molecules, and the involvement of signaling pathways [9,10].

After injury, VECs become activated and produce inflammatory molecules: in particular, monocyte chemoattractant protein-1 (MCP-1), IL-8, intercellular adhesion molecule-1 (ICAM-1), vascular adhesion molecule-1 (VCAM-1), E-selectin, P-selectin, and other inflammatory factors. Via these molecules, the recruiting of immune cells, such as lymphocytes and monocytes, is implemented. They attach to VECs, enter the vessel wall, and initialize inflammation. A wide range of inflammatory mediators are released by T and B cells, dendritic cells, vascular smooth muscle cells (VSMCs), and VECs, which results in the activation of cytokines, chemokines, bioactive lipids, and adhesion molecules that initiate the local inflammation and progression of focal AS damages [11]. Lipoprotein accumulation in the intima layer of the vessel wall and massive migration of immune–inflammatory cells such as lymphocytes and monocytes correlate with major histocompatibility complex (MHC) class II molecule expression in diffuse intimal thickening (DIT), which is the earliest pre-lesional stage in AS development [12]. The monocytes then differentiate into macrophages (MPhs). Among all the immune cells, monocytic MPhs are the major contributors to AS lesions, as they promote arterial inflammation, produce reactive oxygen and nitrogen, secrete a variety of pro-inflammatory mediators in response to stimulation, phagocytize mLDL-C, and then develop into foam cells [13].

### 2.2. M1 and M2 MPhs Polarization

Monocytes recruited into vessel walls can differentiate into MPh of various subsets with different phenotypes and functions depending on the specific stimuli. MPhs differentiate into pro-inflammatory (M1) and anti-inflammatory (M2) cell populations emitting the corresponding pro- and anti-inflammatory cytokines. M1 macrophages are pro-atherogenic, while M2 macrophages may promote tissue repair and have anti-inflammatory properties [14]. Under normal conditions, MPhs respond to damage-associated molecular patterns (DAMPs), pathogen-associated molecular patterns (PAMPs), or mLDL-C, which results in a pro-inflammatory immune response and the release of appropriate cytokines. Following this, immuno-tolerance is developed, and upon that development, MPhs do not respond to previous stimuli, meaning that inflammation does not progress. However, in the case of mutations in the MPhs’ DNA, the cells lose the immuno-tolerance, as a result of which they react to external stimuli and begin to intensively secrete pro-inflammatory cytokines, leading to chronic inflammation and CVD [15].

M1 macrophages are stimulated by Th1 cytokines, such as GM-CSF and IFNγ, as well as lipopolysaccharide (LPS), fatty acids, and HMGB1 (a chromatin protein that interacts with transcription factors, organizing DNA and regulating transcription). M1 MPhs are regarded as pro-atherogenic, since they produce high levels of TNF-α, NO, IL-1β, IL-6, IL-12, and IL-23, which play a crucial role in tissue destruction. M1 expresses pro-inflammatory transcription factors, such as nuclear factor-κB, signal transducer, and activator of transcription (STAT) 1. On the contrary, M2 MPhs are polarized by Th2 cytokines such as IL-4, IL-10, IL-13, and M-CSF. M2 MPhs produce anti-inflammatory cytokines such as IL-10 and TGF-β to restrain inflammation and accomplish tissue repair [16]. MPhs have bilateral capabilities to overthrow pathogens even as they repair inflammation-generated vessel disturbance. Through M1/M2 MPhs differentiation, balance manipulates the conditions of the vessel wall in inflammation or injury [17]. The different MPh subsets are intimately involved throughout atherosclerosis progression and in models of regression. Elucidation of the role of the balance between pro-inflammatory and anti-inflammatory factors in atherogenesis will allow the development of new pharmacological and gene treatments for AS and CVD [18].

### 2.3. Vascular Smooth Muscle Cells (VSMC)

VSMCs also play an important role at all stages of AS development. During atherogenesis, they migrate from the media into the intima, wherein they undergo phenotypic conversion and restructuring into several cell types, such as Mphs, mesenchymal stem cells, osteochondrogenic-like cells, myofibroblast-like cells, and proliferative synthetic cells, that synthesize extracellular fibrous matrix and thus provide added value to AS progression [19]. Upon mLDL-C consumption by VSMC, they transform into MPh-like cells, expressing the scavenger receptor CD68 and other specific MPh markers, such as CD11b and galectin-3. VSMCs can switch to different phenotypes, demonstrating their role in AS progression. Transformed VSMCs are characterized by increased secretion of pro-inflammatory cytokines and exosomes that can prompt osteopontin expression and release calcium sediments. VSMCs can transubstantiate into endothelial-like cells characterized by CD31 expression influenced by shear stress. The main function of VSMC in the formation of AS plaques is the production of fibronectin, collagen 1 alpha 1, and proteoglycans [20].

### 2.4. CHIP

The accumulation of somatic mutations with age is a direct consequence of the constant effects of stress on cells, as well as exposure to DNA-damaging chemical agents. Mutations, called “driver mutations”, provide cells with a selective advantage. Chronic inflammation or, for example, constant exposure to certain stimuli allow cells carrying the corresponding driver mutations to become dominant in the population. Therefore, when a single ancestor cell of a positively expanded clone receives additional driver mutations, cancer cells appear. Remarkably, clonal expansion occurs even in tissues that appear normal. Among the different forms of clonal expansion, clonal hematopoiesis (CH) has been most intensively studied. The term CHIP (CH of indeterminate potential) refers to the presence of at least one driver mutation in peripheral blood hematopoietic cells without hematological malignancy. The precursors of these cells are hematopoietic stem cells (HSCs), which have acquired somatic driver mutations. As in stem cells of other tissues, the accumulation of somatic mutations in HSCs occurs in an age-dependent manner. CHIP is a significant risk factor for the development of several different pathologies, such as acute myeloid leukemia (AML), myelodysplastic syndrome (MDS), and myeloproliferative neoplasms (MPN), as well as cardiovascular diseases.

According to available data, diabetes, hypercholesterolemia, and other metabolic diseases can provoke changes in HSPC function and monocytosis. The differentiation of inflammatory monocytes is one of the central events in the pathogenesis of atherosclerosis. In 2014, there was evidence that CHIP-associated mutations are also associated with the risk of cardiovascular disease. A 2017 study showed that CHIP, together with mutations in the DNMT3A, TET2, ASXL1, or JAK2 genes, was associated with an increased risk of cardiovascular disease. Several studies in LDLR knockout mice suggest a causal relationship between the activation of inflammation in macrophages due to CHIP-associated mutations and the pathogenesis of cardiovascular disease [21].

## 3. Atherogenesis Markers

### 3.1. C-Reactive Protein (CRP)

CRP is an important indicator of the inflammation process and AS progression in the vessel walls [22]. An increase in the CRP level is also a significant risk factor for CVD [23]. With inflammation, pentameric CRP dissociates into five subunits, and the monomeric CRP (mCRP) acquires proatherogenic properties. mCRP activity enhances up to 1000-fold in response to acute inflammation. Under these circumstances, CRP can be considered a biomarker of inflammation and CVD. It participates in recruiting lymphocytes and monocytes, releases a variety of cytokines, such as Interleikin-6 (IL-6) and TNF-α, and also takes part in switching monocytes and T cells to pro-inflammatory phenotypes. Moreover, it is implicated in modified LDL (mLDL) ingestion by macrophages. Thus, CRP is closely associated with many factors involved in the atherogenesis process. mCRPs were found in atherosclerotic lesions from the human aorta, carotid, and coronary arteries. Thus, CRP involvement in atherogenesis has been established [24]. The bloodstream CRP grade of concentration is a superb instrument for diagnosis and controlling administration of patients with atherosclerosis [25]. CRP is absent in vessel walls unaffected by AS, but the CRP content rises in atheromatous tissues and increases progressively as AS develops. Accordingly, it possesses the ability to predict the risk of AS and CVD [26].

### 3.2. Cytokines and Chemokines

Cytokines and chemokines are the soluble factors that can activate various cells involved in AS pathogenesis. Pro-inflammatory cytokines stimulate atherogenesis progression. On the other hand, anti-inflammatory cytokines inhibit inflammation and have a beneficial effect on the disease. Cytokines could be divided into several classes: interleukins (ILs), tumor necrosis factors (TNFs), interferons (IFNs), transforming growth factors (TGFs), colony-stimulating factors (CSFs), and various chemokines. They are produced by different cells, such as T helper cells, monocytes, MPhs, and B cells. T helper cells localized in vascular walls were subdivided into two categories: Th1, producing pro-inflammatory cytokines, such as TNF-α, IL-1β, IL-6, IL-12, IL-18, and IL-23; and Th2, producing anti-inflammatory cytokines, such as IL-4, IL-10, IL-13, IL-19, IL-33, IL-35, and M-CSF. During AS progression, the array of cytokines produced by Th cells may be dynamically changed. An aggravated pro-inflammatory cytokine production leads to AS progression and promotes CVD [27]. Immunohistochemical localization of the inflammation markers in the human aortic wall during atherogenesis revealed the differences in localization of pro-inflammatory cytokines (TNF-α) and anti-inflammatory chemokines (CCL 18). At the initial stages, the association of TNF-α with immune and smooth muscle cells and diffuse distribution of CCL 18 chemokine, as well as a significantly increased expression of CCL 18 marker in atherosclerotic lesions and their translocation from the upper zone of the intima to tunica media, were demonstrated. Simultaneously, the reduction in the level of the pro-inflammatory cytokine TNF-α was recorded with the development of atherosclerosis [28].

### 3.3. Interleikin-6

This inflammatory cytokine synthesized by activated macrophages and T cells represents an important component of the immune system. It functions as a B cell stimulatory factor and causes terminal differentiation of B-lymphocytes to plasma cells. IL-6 controls the course of the inflammatory process. Consequently, IL-6 could be used as a marker to predict CVD [29].

### 3.4. Major Histocompatibility Complex (MHC) Molecules

MHC class II—HLA-DR antigen is normally expressed only by cells of the immune system. These antigens can be specifically recognized by CD4+ Th cells that facilitate activation of MPhs and B cells releasing cytokines, such as interleukins and interferon (IFN)-γ, at the site of infection of AS lesions [30]. The atherosclerotic plaques, however, are composed of a heterogeneous population of cells, which includes VECs, VSMC, MPhs, and T cells. In atherosclerosis-transformed tissue, the majority of all these cell types express MHC class II—HLA-DR. In contrast, very few HLA-DR-positive cells were found in normal human arteries, so MHC may be presented as an AS marker [31].

### 3.5. Cell Adhesion Molecules (CAMs)

CAMs are a subset of cell surface proteins involved in the binding of cells with other cells or with the extracellular matrix. They include isoforms such as ICAM-1, VCAM-1, E-selectin, and P-selectin. CAMs play a significant role in AS development [32]. They are involved in numerous processes in the organism, including cell identification, mobilization, signal transduction, and differentiation. They also intermediate inflammation and immune responses and participate in the AS plaque progression [33]. The expression of CAMs is accomplished in VECs, and leukocytes in the bloodstream reciprocate inflammatory stimuli at the beginning of AS development. With that, CAMs mediate the recruitment of inflammatory cells from the circulation and their transendothelial migration [34]. All isoforms of cell adhesion molecules were identified in areas of atheromatous lesions [35]. All these results suggest that CAMs can be used for therapeutic purposes [36].

## 4. Lipoprotein Hypothesis

One of the most common and well-known causes of AS development is an increased and modified low-density lipoprotein cholesterol (mLDL-C) infiltrating the vessel endothelium, which triggers intimal accumulation and retention of apolipoprotein B (apoB)-containing LDL in focal areas of arteries, followed by inflammatory response [37,38,39]. Among the several subclasses of LDL particles, which have various dimensions and compactions, the small dense LDL (sdLDL) particles present a maximal atherogenic capability compared to the rest of the LDL subcategories, so they are superior markers for diagnosis of cardiovascular disease [40].

mLDL, such as oxLDL, tends to aggregate in the intima of blood vessels. These aggregates stimulate leukocyte recruitment and phagocytosis of macrophages and contribute to the secretion of pro-inflammatory cytokines, accumulation of intracellular cholesterol, and foam cell formation [8]. Several genes affinitive with signaling pathways, in particular *F2RL1*, *EIF2AK3*, and *IL15*, are responsible for encoding inflammatory molecules and regulating the interaction of mLDL with macrophages [41,42].

## 5. Relationship between CRP and mLDL

There is much evidence concerning the relationship between CRP and mLDL in the atherosclerotic process [43]. Elevation of both CRP and mLDL levels predicts the risk of CVD. Notably, the CRP level is a major indicator of cardiovascular events compared to the LDL-C level in predicting CVD [44]. It was established that CRP is capable of specifically binding to mLDL through calcium participation [45]. These two associated compounds are present in AS plaques and indicate the excessive risk of CVD [46]. The stages of AS progression upon LDL-C modification are as follows: after modification and oxidation of LDL-C in blood, plasma mLDL (oxLDL) is converted into atherogenic lipoprotein. A cytotoxic effect of mLDL on endothelial cells, the stimulation of chemotaxis, and the migration of monocytes into the intima with their subsequent differentiation into macrophages occur. Then, macrophages ingest m-LDL (by scavenger receptors) and transform into foam cells. m-LDL accumulates in fatty streaks, cytokines and chemokines are released, and the unfolding of the inflammatory response cascade occurs. Hyperplasia of smooth muscles occurs, as well as extracellular fibrous matrix production and the formation of lipofibrous plaque [47].

## 6. Oxidative Transformation Hypothesis

Many factors contributing to LDL modification in the bloodstream and vessel walls exist. Upon modification, LDL transforms into mLDL, changing size and physicochemical characteristics. Among these changes, LDL oxidation is believed to be the primary modification in AS development. The risk factors contributing to oxLDL formation include, for example, genetic predisposition, smoking, infection, hypertension, and diabetes. The pertinent enzymes that participate in LDL oxidative modification include NADPH oxidase, lipoxygenases, xanthine oxidase, myeloperoxidase, reactive oxygen species (ROS), and endothelial nitric oxide synthase (eNOS) [48].

OxLDL can bind to scavenger receptors (SR), including SR-A1, SR-A2, and LOX-1. OxLDL also upregulates the expression of the LOX-1 receptor on VECs and manages the activation of these cells. Moreover, oxLDL gives rise to the growth and displacement of VSMCs, monocytes, and fibroblasts. Additionally, oxLDL promotes ROS production, which, when in excess, triggers oxidative stress [49]. OxLDL can initialize the inflammation process through the activation of macrophages and other immune cells. As a result of oxLDL focal infiltration, foam cell formation and fatty streaks development occur, followed by atherosclerotic plaque formation after endothelial dysfunction, monocyte transmigration to the vessel wall, proliferation and displacement of VSMCs, and platelet activation [50].

## 7. Shear Stress

One of the most important factors in maintaining endothelial homeostasis under normal physiological conditions is the friction force acting on endothelial cells, otherwise known as hemodynamic shear stress. Considering endothelial dysfunction and the effect of blood flow on it, one can speak of two types of blood flow, stable laminar flow and disturbed flow, since endothelial cells respond differently to these types of flow both in vivo and in vitro. Laminar flow, which is exerted by steady laminar shear stress, is atheroprotective, while disturbed flow promotes atherosclerosis. Emerging data have provided new insights into the cellular mechanisms of flow-dependent regulation of vascular function, which leads to cardiovascular events such as atherosclerosis, atherothrombosis, and myocardial infarction [51]. Shear stress eventuates in vascular endothelium sites because frictional force arises in blood circulation predominantly in areas of artery curves, branching, and bends due to the mechanical impression. It exerts a significant impact on inflammation triggering, vascular dysfunction, and AS. Immune cells swoop down to these damaged endothelium areas and infiltrate into the intima, transforming into macrophages that actively swallow up mLDL [52]. The mechanisms behind shear stress functioning are not completely unraveled. For a better understanding of these mechanosensitive signaling pathways (MSP) in arteries, systems biology approaches are employed, including transcriptome, proteome profiling, and functional screening platforms [53].

The vascular endothelium has specialized receptors that specify blood flow and convey signals with the aid of MSP to responding compounds that result in atherogenic development. In many clinical observations, it has been established that atherosclerotic distinctive features revealed themselves predominantly at arterial inflections that represent the areas shown to have altered blood flow. In these endothelium areas, the inflammatory process is initiated, the release of nitric oxide is ramped down, barrier function is reduced, and adhesive power and proliferative efficiency are increased. These endothelial peculiar features may elucidate the preferred advent of atherogenesis in arterial sites with disturbed flow [54].

## 8. Hypothesis of Vessel Endothelium Dysfunction

Endothelial dysfunction is caused by a wide range of risk factors, such as high levels of mLDL (oxLDL), dyslipidemia, insulin resistance syndrome, diabetes, arterial hyperglycemia, genetic defects, dysregulated renin–angiotensin system, hypertension, and shear stress [55].

There are several pathological occurrences of endothelial dysfunction, such as nitric oxide synthase dysfunction and activation of inflammatory mediators, including oxLDL, cytokines, and other molecular patterns. Pro-inflammatory cytokines are the key figures in atherogenesis, imparting the AS plaque formation in affected vessels. The switching-in of multiple signaling pathways, NF-kβ in particular, which controls the DNA transcription, triggers the overproduction of adhesion molecules, selectins, and chemokines that promote monocyte migration to the vascular intima, VECs apoptosis, flow-sensitive microRNA regulation, activation of coagulation pathways, and downregulation of thrombomodulin, leading to AS progression [56]. This hypothesis proposes that the main causes of AS development are endothelial dysfunction, which is accompanied by insufficient NO production, and substances such as prostacyclin, hyperpolarizing factor, and endothelin [57]. Taking together, VECs play an ultimate role in the process of AS and CVD development through their regulatory functions.

Nitric oxide (NO) is a product of transforming L-arginine to L-citrulline by the enzymatic action of NO synthase (eNOS) in the endothelium [58]. In the case of atherogenesis, one of the main NO functions is vascular endothelium-dependent relaxations (EDR), which is accomplished during NO liberation from the vascular endothelium, and so this agent acts as an endothelium-derived relaxing factor (EDRF) [59]. NO, as EDRF, inspires guanylate cyclase of the vascular smooth muscle that leads to an escalation in cGMP activating relaxation. eNOS represents an anti-atherogenic substance, and insufficiency of eNOS leads up to atherogenesis triggering. AS is concerned with an EDR malfunction, which comprises the decrease in NO bioavailability. The main cause of this event in AS is superoxide excreted through the dysfunction of eNOS as a result of all sorts of pathological conditions. Consequently, EDRF activity is blocked by superoxide. In addition, defective eNOS may disable EDR, and it can also damage atherosclerotic vessels [60].

## 9. Homocysteinemia

Homocysteine is a derivative of the amino acid methionine resulting from the metabolic process. The increase in blood homocysteine proceeds from B-vitamin limitation, genetic factors, and some kinds of drugs. Hyperhomocysteinemia initiates oxidative stress, endothelial dysfunction, an increase in arterial pressure, AS, and thrombosis. Homocysteine is the AS causative agent that acts as an independent risk factor. An elevated homocysteine level in circulation increases CVD risk nearly two-fold [61,62]. Homocysteine contributes to vascular inflammation and atherosclerosis acceleration driven by excessive emission of inflammatory factors, especially IL-1β [63].

## 10. AS Induction by Diabetes Mellitus

Diabetes mellitus is triggered by disorders of carbohydrate metabolism. The disease is characterized by a high blood glucose level (hyperglycemia) due to either the lowering of insulin production by the pancreas or the insulin resistance of target cells. Cardiovascular disease and AS associated with diabetes are dependent not only on hyperglycemia but also on alterations in lipids, changes in hormones in addition to insulin, and a pro-inflammatory state [64]. Diabetes-associated dyslipidemia is a key element of influence on atherogenesis. Alteration of the blood lipid composition in diabetes is linked to the increased formation of atherogenic lipoproteins [65]. Elevated glucose levels, insulin resistance syndrome, dyslipidemia, oxidative stress, inflammation, and other alterations have a great impact on atherogenesis. Concordantly, diabetes mellitus exerts an influence on the atherogenic process through its relationship with chronic inflammation [66].

## 11. Bacterial and Viral Infections (“Infection Hypothesis”)

An idea proposed by William Osler at the beginning of the 20th century that infections also play a role in atherosclerosis [67] has been backed up by an increasing body of evidence at the end of the 20th and beginning of the 21st century. Since the end of the 1970s, the possible involvement of microorganisms and viruses in AS development has been widely discussed in the literature. In several studies, it has been demonstrated that animals infected with various pathogens showed significant arterial changes, compared with the control groups of animals. Microscopically, these lesions were characterized by intimal thickening, which formed fibrous caps that overspread the atherosclerotic change loci [68]. Infectious agents also influence lipids metabolism and promote cholesterol accumulation in the arterial walls [69]. At that time, there were no direct pieces of evidence for infectious agents involved in the atherogenic process. Since then, much research has been carried out suggesting that bacteria and viruses entail cellular and molecular changes in vessel walls. The serological evidence of antibodies to some groups of bacterial and virus antigens present in patients with chronic coronary heart disease and acute myocardial infarction, which have been absent in the control group of patients, was demonstrated. Moreover, a co-relation of IgG antibodies against these pathogens with AS of large arteries has been elicited [69]. Beyond that, the pathogens, such as *Chlamydia pneumonia*, *Helicobacter pylori*, human cytomegalovirus (HCMV), Epstein–Barr virus (EBV), and herpes simplex virus (HSV), have been identified in human arterial lesion samples through the use of histopathological, immunocytochemical, and ultrasonographic imaging studies, apart from seroepidemiological assays. Pathogens can transform VSMC, followed by migration of these cells in AS lesions. Various vascular cell types, such as VSMC, VECs, and leukocytes, presented in AS lesions can produce and react to cytokines [70]. The hypothesis on the variety of atherogenic-associated pathogens’ existence gradually became more and more popular [71].

Several studies have been carried out to identify bacterial DNA in biopsy samples from AS lesions of patients using various methods. The list of identified microorganisms can be found in Table 1. However, the microbiome composition in atheromatous regions may significantly differ from patient to patient. Therefore, it seems difficult to determine which of the identified bacterial species are involved in atherosclerotic plaque development. Further, more precise research methods are needed to solve this problem.

The presence of a wide range of bacterial species in atheromatous lesions may appear to have a greater effect on vascular tissues than individual species. The detection of numerous types of bacteria in vascular atheromas by different methods does not allow us to make an unambiguous conclusion that pathogens can be an etiological factor that triggers atherogenesis in the vessels. However, the microbiome may be a concomitant factor capable of exacerbating and/or accelerating inflammation and disease progression [76]. A large number and variety of bacterial types in areas of atherosclerotic vascular lesions may be associated with increased resistance of the microbiome to antibiotics, immune cells, and other factors due to the biofilm formation and buildup of an insoluble exopolymer matrix [77]. Thereby, a bacterial biofilm structure may complementarily be responsible for inflammation in the pathogenesis vessel walls and cause the AS development [74].

Risk of infection-induced AS development depends at least in part on the amount of atherogenic pathogens in the infected patient, as well as on their responsivity to the lesion effects of pathogens; in other words, whether the host is able to create an immune response and thus control the infectious process and a pathogen-induced atherogenesis [78]. It was also established that infection was associated with an increase in the content of fibrinogen, leukocytes, clotting factor, cytokines, and elevated levels of CRP in the blood, as well as a clear change in the functioning of the vascular endothelium, monocytes, and macrophages. Moreover, it is accompanied by CVD and acute ischemic symptoms [79]. “Infection hypothesis” does not disable the contribution of all other risk factors for atherosclerosis. As a matter of fact, infections may functionalize through the mediation of or in consort with all other factors, such as mLDL-C, homocysteinemia, shear stress, endothelial dysfunction, action of free radicals, diabetes mellitus, and decreased nitric oxide level. Infection caused by pathogenic bacteria or viruses may be responsible for inflammatory reaction cascades in areas of atherosclerotic lesions in arteries, and, simultaneously, any of the risk factors may also lie behind this inflammation and atherogenesis. In Table 2, we summarize the involvement of selected pathogens in atherosclerosis development.

## 12. Oral Cavity Pathogens

A huge variety of bacteria inhabit the oral cavity [80]. It has been shown that some of the oral microbiota bacterial species demonstrate a pronounced correlation with atherosclerotic vascular lesions and an increase in blood cholesterol levels. In particular, Koren et al. noted bacteria from the genera Chryseomonas, Veillonella, and Streptococcus. These bacteria were highly numerous both in the oral cavity and in the AS plaques simultaneously in the same patient [89]. A number of studies have presented data on the close relationship between chronic periodontitis (CP) and CVD as well as CP’s correlation with blood cholesterol levels [90]. Detection of CP-causing bacteria in vessel wall areas impaired by AS was performed particularly by using PCR procedure. Moreover, DNA–DNA hybridization was also administered to estimate the periodontal pathogens in subgingival areas from CP patients and control group. The number of bacteria colonizing areas of the vessel walls affected by AS in patients with periodontitis significantly exceeded those in patients without CP. These data suggest the implication of periodontal bacteria in atherogenesis. A great number of bacterial species were identified in atherosclerotic lesion samples isolated from CP patients. Among them, there are many established periodontopathogen bacteria, such as *Porphyromonas gingivalis*, *Tannerella forsythia, Treponema denticola*, *Aggregatibacter actinomycetemcomitans*, *Prevotella intermedia*, and *Fusobacterium necrophorum* [91]. Comparison of results obtained from patients with and without CP led to the conclusion that these bacteria are implicated in AS pathogenesis [92]. The comparative quantification of periodontal pathogens (such as *Aggregatibacter actinomycetemcomitans, Porphyromonas gingivalis, Campylobacter rectus,* and *Tannerella forsythia*) in subgingival samples, blood, and vessel walls from patients with AS disorders was performed using two molecular identification methods (nested PCR and quantitative PCR). The results confirm the assumption that bacteria are able to move from the periodontal area into the vessel walls with AS lesions [93].

## 13. Gut Microbiota

Some recent research has contributed to understanding the relationship of gut microbiota with inflammation and atherogenesis. Thus, Brandsma et al. (2019) conducted experiments on the transplantation of fecal microflora from Caspase1-/-(Casp1-/-) mice to Ldlr-/-mice and revealed AS acceleration, an elevated level of blood leukocytes, pro-inflammatory cytokines, and neutrophil accumulation in atherosclerotic plaques of Ldlr-/-mice, compared to the control group [94]. AS progression and CVD are caused specifically by disarrangements in the intestinal microflora and gut dysbiosis [95]. An increase in the gut bacteria transmigration into the circulating blood, as well as bacterial metabolic products, leads to the emergence and intensification of the inflammatory process in the vessel wall. Some of the bacterial metabolites released into the bloodstream, such as trimethylamine and trimethylamine N-oxide, short-chain fatty acids, and secondary bile acids, are involved in AS and CVD progression [96]. In several studies, an obvious relationship was noted between abnormal metabolism of the gut microbiome and the progression of inflammation and AS. The mechanisms of gut microbiota’s impact on atherogenesis appear to comprise the initiating of inflammation processes via alterations of lipid metabolism in adipocytes, macrophages, and VECs, stimulating insulin resistance, and producing trimethylamine-N-oxide. The triggering factor of lipid metabolism abnormalities could be bacterial LPS delivered from the intestine to the bloodstream via chylomicron transfer [97]. It has been established that LPS can increase the morbidity and mortality of AS-related CVD. Particularly, LPS enlarges the intimal layer and facilitates lipid accumulation through the mobilization of the TLR4-NF-κβ pathway. Moreover, LPS promotes MCP-1 generation from activated adventitial fibroblasts, followed by monocyte capture to the vessel wall, accumulation of lipids in macrophages, and foam cell formation [98]. Concerning changes in the bacterial composition of the gut microbiome affecting CVD development, it has been observed that a decrease in Bacteroidetes and an increase in Firmicutes, the most representative groups of bacteria in the gut, have an appreciable impact on atherogenesis and CVD occurrence [99].

## 14. Molecular Mechanisms of Infection-Stimulated Atherogenesis

In the first stage, infectious agents directly damage VECs, which leads to increased ROS production. Elevated ROS production by polymorphonuclear neutrophils at the inflammation area causes oxidation of cellular signaling proteins, such as tyrosine phosphatases, and promotes the migration of inflammatory cells through the endothelium [100]. This not only destroys bacterial and viral pathogens but leads to tissue damage. Moreover, ROS can promptly bind to NO, forming reactive nitrogen species (RNS). RNS causes nitrosative stress, which supplements the pro-inflammatory charge of ROS [101]. Pathogen-induced oxidative stress is one of the most important factors causing CVD. ROS, localized in VECs, plays a key role in provoking an inflammatory response, cell apoptosis, and promoting NF-κb signaling [102]. Inflammation in vascular walls generated by infection is the first stage of AS. Inflammation causes VECs to induce heat shock protein 60 (Hsp60) secretion. T-cell-mediated autoimmune reaction against Hsp60 is the initiating episode in atherogenesis, as suggested by Wick G. et al. in 1995. The inflammatory stage that follows progresses with atherosclerotic lesions and goes through all the classical pathological effects [103]. Hsp60 is a molecular chaperone, participating in protein folding and impeding the magnification of misfolded proteins. In the vascular wall, Hsp60 has a preserving function under physiologic conditions. In the case of any pathology, though, Hsp60, localized on the VECs surface, triggers autoimmune processes [104]. The protein GroEL is also a member of the chaperonin family and is found in many bacteria. It is very close in structure and function to Hsp60. GroEL can enhance monocyte adhesion by elevating the expression of ICAM-1 and VCAM-1 in VECs. Moreover, GroEL forced an increase in oxLDL uptake, which depends on the elevated LOX-1 expression. Furthermore, GroEL can interrelate with TLR4 and thus trigger atherogenic events in vessel walls. Accordingly, bacterial GroEL may contribute to CVD by influencing TLR4 expression [105]. TLRs are representative of pattern recognition receptors (PRRs) that are involved in immune processes by identification of pathogenic bacteria and viruses that come across the blood vessel walls. Accordingly, TLRs perform the function of host cells protecting from pathogens [106]. Apart from pathogen recognition, TLRs have numerous other functions, such as coordination between innate and adaptive immunity, regulation of cytokine production, cell proliferation, and supporting survival of the organism in adverse conditions [107]. After TLRs’ sensitization, they are also directly involved in the AS progression caused by infectious agents. The participation of these receptors in the atherogenesis-associated processes suggests the feasibility of using them as targets for the therapeutic approaches developed to prevent CVD [108].

A significant contribution to the stress state of VECs is made by the overproduction of pro-inflammatory cytokines and adhesion molecules induced by infectious pathogens that promote leukocyte and monocyte migration and infiltration into the vessel wall intima [109]. Cytokines comprise several classes, such as interleukins (IL), chemokines, colony-stimulating factors (CSF), tumor necrosis factors (TNF), interferons (IFN), and transforming growth factors (TGF). Cytokines are involved in all stages of atherosclerosis, and all cells implicated in atherogenesis are capable of cytokine production [110]. These molecules perform a dual function. Pro-inflammatory cytokines facilitate AS development, while anti-inflammatory cytokines (IL-4, IL-10, IL-13, TGF-β) display antiatherogenic properties. Pro-inflammatory cytokines, such as TNF-α, IL-1, IL-12, IL-18, CD40L, M-CSF, and IFN-γ, modify the endothelium’s functioning, which leads to the loss of barrier function and intensification of the leukocytes and monocytes’ influx into the vascular walls. Leukocytes that arrive at the intima of the vascular wall transform into macrophages under the influence of local cytokines. IFN-γ can promote foam cell formation via initiation of scavenger receptors that mediate uptake of mLDL and subsequent conversion of macrophages to foam cells. Then and there, IFN-γ binds the two global body functions: immunity and lipid metabolism [111].

## 15. Cytokine-Associated Signaling Pathways

An NF-κβ signaling pathway is one of the highest relevance in the regulation of innate immunity and inflammation processes. NF-κβ is an ancient protein complex operating as a transcription factor that is upregulated in response to a variety of detrimental stimuli, such as inflammation [112]. It was established that NF-κβ is forced into lipid metabolism and atherogenesis processes, including foam cell formation, vascular inflammation, the proliferation of VSMCs, vessel wall calcification, and plaque development [113]. There is a growing body of evidence allowing consideration that NF-κβ plays a substantial role in all stages of atherogenesis through engagement genes, membranes, protein complexes, cytokines, chemokines, and hormones. Therefore, the NF-κβ signaling pathway may serve as a target for therapeutic approaches to discourage an inflammatory process in the blood vessel walls [114]. This pathway is upregulated in response to pro-inflammatory cytokines generation, which results in the TLRs’ activation by the pattern recognition of pathogen-associated molecular patterns (PAMPs). Activation of the NF-κβ pathway plays a fundamental role in the process of inflammation, which is carried out by regulating the expression of genes encoding growth factors, VCAM-1, E-selectin, IL-1, IL-6, IL-8, tissue factor, plasminogen activator inhibitor (PAI)-1, cyclooxygenase (COX)-2, and iNOS. Further development of atherogenesis processes leads to damage of the vessel walls and vascular cell dysfunction [57].

It was also shown that MCP-1 heightened production is induced by many pathogens. This chemoattractant is designated for the transmigration of monocytes [115]. The elucidation of molecular mechanisms behind the cascade of inflammatory responses associated with chronic infection provides more and more evidence on behalf of the infection hypothesis and the interdependency between infections and CVD [5,72].

## 16. Conclusions

Numerous hypotheses about the causes of triggering the atherogenesis mechanism indicate the extraordinary complexity of this phenomenon. Therefore, it is not reasonable to accentuate any single main contributor to initiating AS progression. However, based on growing evidence, a close relationship has been established between atherogenic CVD and various infections. Thus, the paradigm of the infectious nature of atherogenesis may become prevalent. The assumptions that pathogens become involved in the etiology of atherosclerosis are built on the following factual knowledge: (1) the occurrence of antigens to the particular pathogens in atheromatous degeneration; (2) the presence of DNA or RNA sequences of bacteria and/or viruses in atherosclerotic lesions; (3) the relationship between concrete pathogenic infection and accelerated AS plaque growth; (4) the significant effects on cholesterol metabolism in vascular smooth muscle cells infected with pathogens, resulting in cholesteryl ester accumulation; (5) vascular endothelial cells losing anticoagulant capabilities when infected by a pathogen; (6) increased recruiting of inflammatory cells to a pathogen-infected vascular wall; (7) the ability of infectious agents to stimulate the production of cytokines and other pro-inflammatory factors by vascular and inflammatory cells. Thus, the involvement of pathogenic bacteria and viruses in atherogenesis triggering, considering all the evidence presented, is quite evident. However, two approaches to the pathogen’s action are plausible: either through the direct infection of vascular cells or via the indirect effects of cytokines or any other proatherogenic factors induced by infection.

## Figures and Tables

**Table 1:** Overview of the microorganism variety found in the AS lesions.

Microorganisms	Subjects	Method of Detection	References
*Chlamydia pneumonia*	biopsy samples of patients’ AS lesions	PCR	[72,73]
*Porphyromonas gingivalis*			
*Helicobacter pylori*			
*Aggregatibacter actinomycetemcomitans*			
*Mycobacterium tuberculosis*			
*Enterobacter hormaechei*			
*Chryseomonas*			
*Veillonella*			
*Streptococcus*			
*Bacillus idriensis*			
*Pseudomonas* sp.			
*Delftia* sp.			
influenza A virus			
hepatitis C virus			
cytomegalovirus			
human immunodeficiency virus (HIV)			
*Chryseomonas*	biopsy samples of patients’ AS lesions	pyrosequencing t16S rRNA genes	[74]
*Staphylococcus*			
*Propionibacterium*			
*Burkholderia*			
*Flavobacterium*			
*Pseudomonas*			
*Clostridium*			
*Streptococcus*			
*Acinetobacter*			
*Aggregatibacter actinomycetemcomitans*			
*Pseudomonas* sp.			
*Lactobacillus rhamnosus*			
*Neisseria polysaccharea*			
*Waddlia chondrophila*			
*Staphylococcus* species	biopsy samples of patients’ AS lesions	fluorescence in situ hybridization	[75]
*Proteus vulgaris*			
*Klebsiella pneumoniae*			
*Streptococcus* species			
*Chlamydia* species			

**Table 2:** Contribution of the selected pathogens to atherosclerosis development.

Microorganism	Effect in Atherosclerosis	References
*Chlamydia pneumoniae*	triggers immune response in VECs, VSMC, monocytes, and macrophages	[80,81]
	triggers the transformation of monocytes to macrophages, followed by foam cell derivation via chlamydial LPS	
*Porphyromonas gingivalis*	triggers oxidative stress, VECs disfunction, inflammation, and initiating of NF-κβ signaling	[82,83]
	suppresses production of anti-inflammatory cytokines, such as IL-2, IL-4, and IL-10	
	reduces Tregs that are able to inhibit T cell reproduction	
	GroEL heat-shock protein, which significantly increases the atherogenicity	
Human cytomegalovirus (HCMV)	promotes inflammation and VECs damage by initiating the production of adhesion molecules on VECs and their ligands on leucocytes	[84]
	induces VECs apoptosis through a p53-dependent mechanism	
	antibodies against HCMV-derived proteins US28 and UL122 induce the expression of genes encoding growth factors, chemokines, adhesion molecules	
	induces excessive proliferation of SMCs in areas of vessel wall lesions that provoke the intima thickening	
	promotes the NF-κβ signaling pathway	
Human immunodeficiency virus (HIV)	HIV-related proteins (transactivator of transcription (Tat), negative factor (Nef), HIV envelope protein gp120) trigger inflammation and endothelial dysfunction	[85]
	triggers T cell activation	
Herpes simplex virus (HSV)	high level of antibodies specific for HSV in AS patients’ sample	[86]
Influenza A virus (IAV)	elevates blood levels of chemokines and cytokines	[87]
	decreases eNOS expression	
SARS-CoV-2 virus	attacks the immune system, which results in excessive inflammation and perpetuates a vicious cycle of deteriorated endothelial dysfunction that further promotes inflammation	[88]
	activates the NLRP3 inflammasome	
	dysregulates the renin–angiotensin system (RAS)	

## Data Availability

Not applicable.
